# Recurrent metastatic retroperitoneal dedifferentiated liposarcoma: a case report and literature review

**DOI:** 10.1186/s12894-023-01252-3

**Published:** 2023-04-24

**Authors:** Tuming Liao, Wei Du, Xiongcai Li, Shen He, Gangqiang Guan, Herong Zhu, Jiaqiao Wu

**Affiliations:** Department of Urology Surgery, Guangdong Provincial Hospital of Integrated Traditional Chinese and Western Medicine (Nanhai District Hospital of Traditional Chinese Medicine of Foshan City), No. 16, Guicheng South Fifth Road, Nanhai District, Foshan, 528200 Guangdong People’s Republic of China

**Keywords:** Recurrence, Metastasis, Dedifferentiated liposarcoma, Combined organ resection, Targeted therapy

## Abstract

**Background:**

Retroperitoneal liposarcoma (RPLPS), a rare tumor, is often treated using surgical procedures as the first choice for treatment. However, there is no consensus on the scope of surgical resection. In addition, the treatment outcomes of conventional radiotherapy and chemotherapy have not been satisfactory, particularly for specific types of LPS, such as dedifferentiated LPS. In this case study, we present a brief review of other cases of RPLPS, highlighting the selection of surgical scope for RPLPS and related adjuvant treatment for advanced RPLPS.

**Case presentation:**

A case study is reported regarding an extremely rare recurrent and metastatic retroperitoneal dedifferentiated LPS. The primary RPLPS tumor, with a diameter of 20 cm and a weight of 2.5 kg, occupied the whole left abdomen and adhered to the left kidney. A surgical tumor resection combined with a left nephrectomy is performed. During the 6th -month postoperative follow-up examination, we observed the local recurrence of the tumor in the operation area, in addition to multiple metastatic tumors in both lungs. Further, the prescribed 3-month targeted treatment with anlotinib significantly reduced the size of the metastatic pulmonary tumors. However, the recurrent retroperitoneal tumors showed no significant change in size. Eventually, we observed no substantial evidence of tumor progression, with the patient’s condition under control.

**Conclusion:**

The case demonstrated that the postoperative recurrence of widespread RPLPS required R0 resection to cure the disease, considering targeted therapy for advanced RPLPS control.

## Background

Liposarcoma (LPS), a soft tissue tumor, originates from the primitive mesenchymal cells differentiated from adipocytes, accounting for approximately 10–15% of all soft-tissue sarcomas. LPS occurs predominantly in the deep soft tissues of the limbs, in which 12–40% of LPS is localized in the retroperitoneal space, with approximately 35% originating from the perirenal adipose tissue [1, 2]. Notably, patients with retroperitoneal LPS (RPLPS) are mostly asymptomatic until the tumor has enlarged sufficiently to compress surrounding organs. Therefore, the diagnosed tumor has usually developed enormously (more than 15 cm) and started compressing or invading the surrounding organs [3]. More often, surgical intervention is often referred to resect the tumors. Even in the cases of successful resection of RPLPS tumors, most patients still need additional therapeutic modalities, such as secondary surgery, radiotherapy, traditional chemotherapy, or targeted therapy, due to the high level of tumor recurrence with high invasion tendencies [4]. In this study, we report a case of substantial retroperitoneal dedifferentiated LPS, weighing 2.5 kg, occupying the whole left abdomen, and adhering to the left kidney. The tumor was successfully resected along with the affected kidney. After 6 months of surgery, the patient showed not only multiple recurrent metastatic tumors in both lungs but also recurrent tumors in the operation area. To treat the recurrent metastatic tumors, we have employed the targeted treatment with anlotinib, resulting in a significantly decreased tumor load for this patient.

## Case presentation

**Fig. 1:**
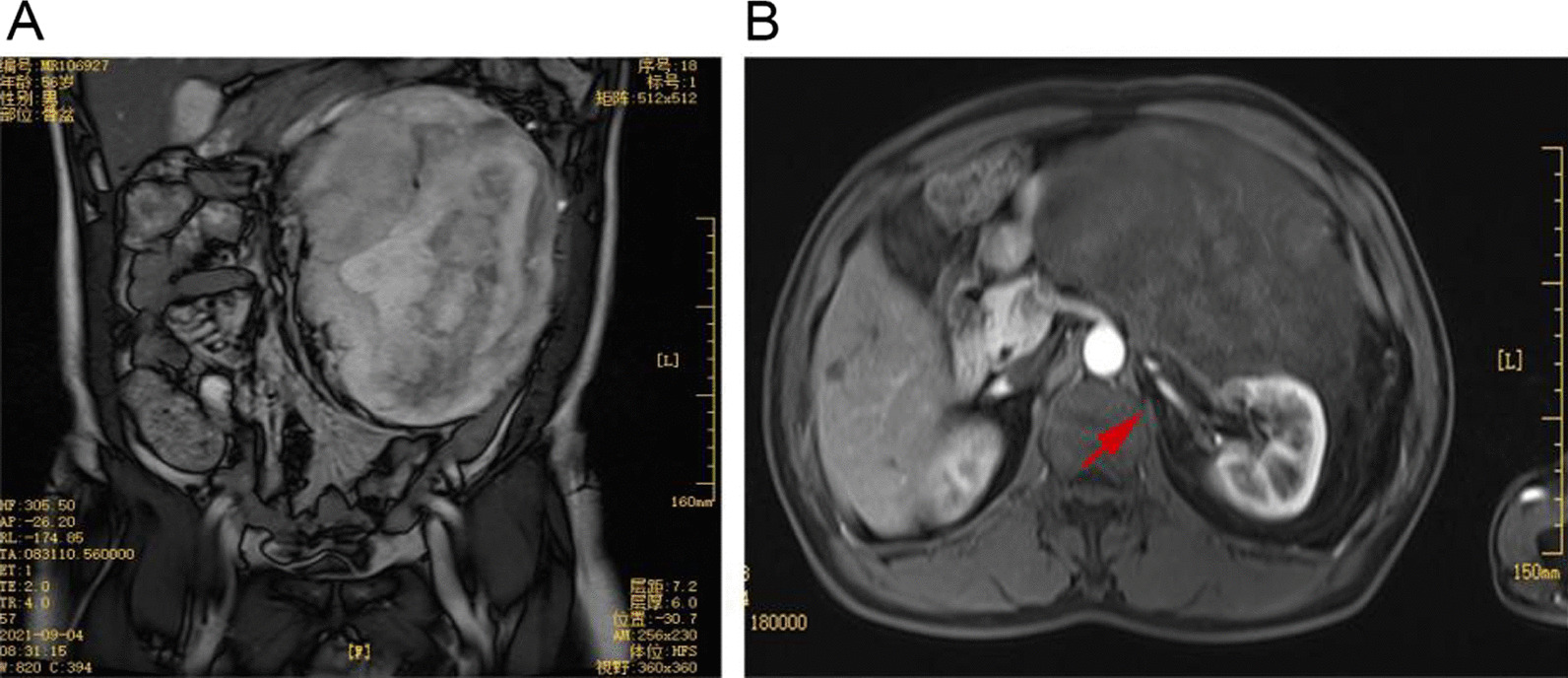
**A** The tumor occupies the entire left abdominal cavity and pushes the visceral organs of the abdominal cavity to the right abdominal cavity. **B** The tumor originates from the retroperitoneal region and surrounds the left kidney. The red arrow indicates the vessels of the left renal hilum closely related to the tumor

Initially, a 56-year-old man complained about left scrotum sagging and swelling. Further, the physical examinations revealed the presence of a sizeable palpable mass throughout the left abdomen. Further, the biochemical laboratory examination resulted in hemoglobin levels of 106 g/L. Magnetic resonance imaging (MRI) examination revealed a colossal mass shadow in the left retroperitoneal cavity. The surrounding tissues and organs were ultimately squeezed and disorganized, especially the structure of the left renal hilum, assuming that they might have been invaded (Fig. 1). Further, no lung abnormality by the chest computed tomography (CT) and no apparent irregularities in other examinations were found. Therefore, the panel of surgeons has decided to operate and resect the tumor.

**Fig. 2:**
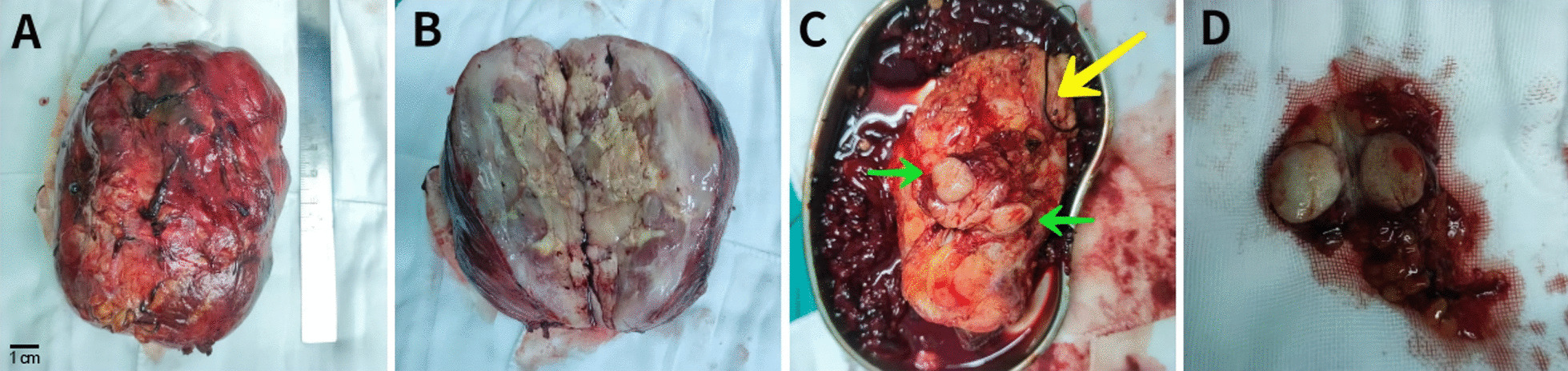
**A** The tumor size indicates an approximate size of 20 * 16 * 10 cm^3^ in size and a weight of 2.5 kg. **B** The appearance of tumor resembles a rotten fish. **C** The yellow arrow indicates the left kidney and the green arrow indicates the tumor outside the capsule of the lower pole of the left kidney. **D** The image shows the seperated removed tumor

**Fig. 3:**
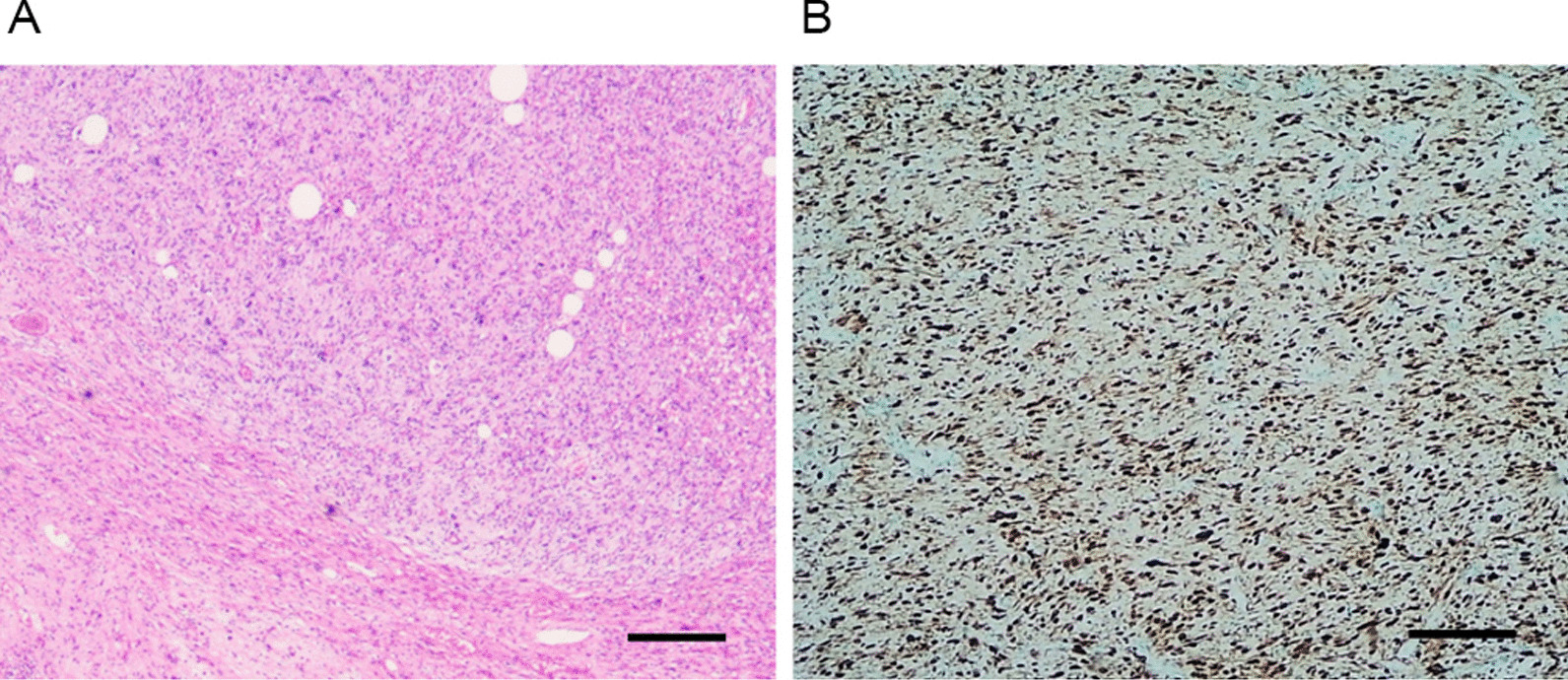
**A** The image shows the pathological section of DDLPS after H&E staining (scale: 100 μm). **B** The image presents the cytoplasmic staining of specific antigens in retroperitoneal DDLPS cells through immunohistochemical staining (scale: 100 μm)

During the open surgery, in the supine position and with a supra-infra umbilical incision, we detected a solid tumor of approximately 3 × 2 cm^2^ in the capsule of the lower pole of the left kidney. Moreover, it was observed that the massive retroperitoneal tumor adhered to the surrounding tissues and organs, especially between the tumor and the left kidney, leading to difficulty in its separation. In addition, the network of blood vessels near the left renal pedicle was surrounded and penetrated deeply into the tumor. Thus, we decided to remove the tumor and the left kidney and clean the surrounding adipose tissue as much as possible. The excision site was approximately in the dimensions of 20 * 16 * 10 cm^3^ and weighed about 2.5 kg (Fig. 2). Further, the histopathological changes were demonstrated using hematoxylin & eosin (H&E) and immunohistochemical stainings of the excised tumor tissues and observed under Olympus BX53 microscope equipped with Golden Domain LIR System V1.0.22.526 (Olympus Corp., Tokyo, Japan). These microscopic observations showed that the left lower pole extracapsular tumor and the giant retroperitoneal tumor were of dedifferentiated LPSs. Remarkably, no tumor infiltration was found in the left kidney, ureter, and blood vessels. Moreover, the immunohistochemistry (IHC) analysis results indicated the cells were MDM2-positive (Fig. 3).

**Fig. 4:**
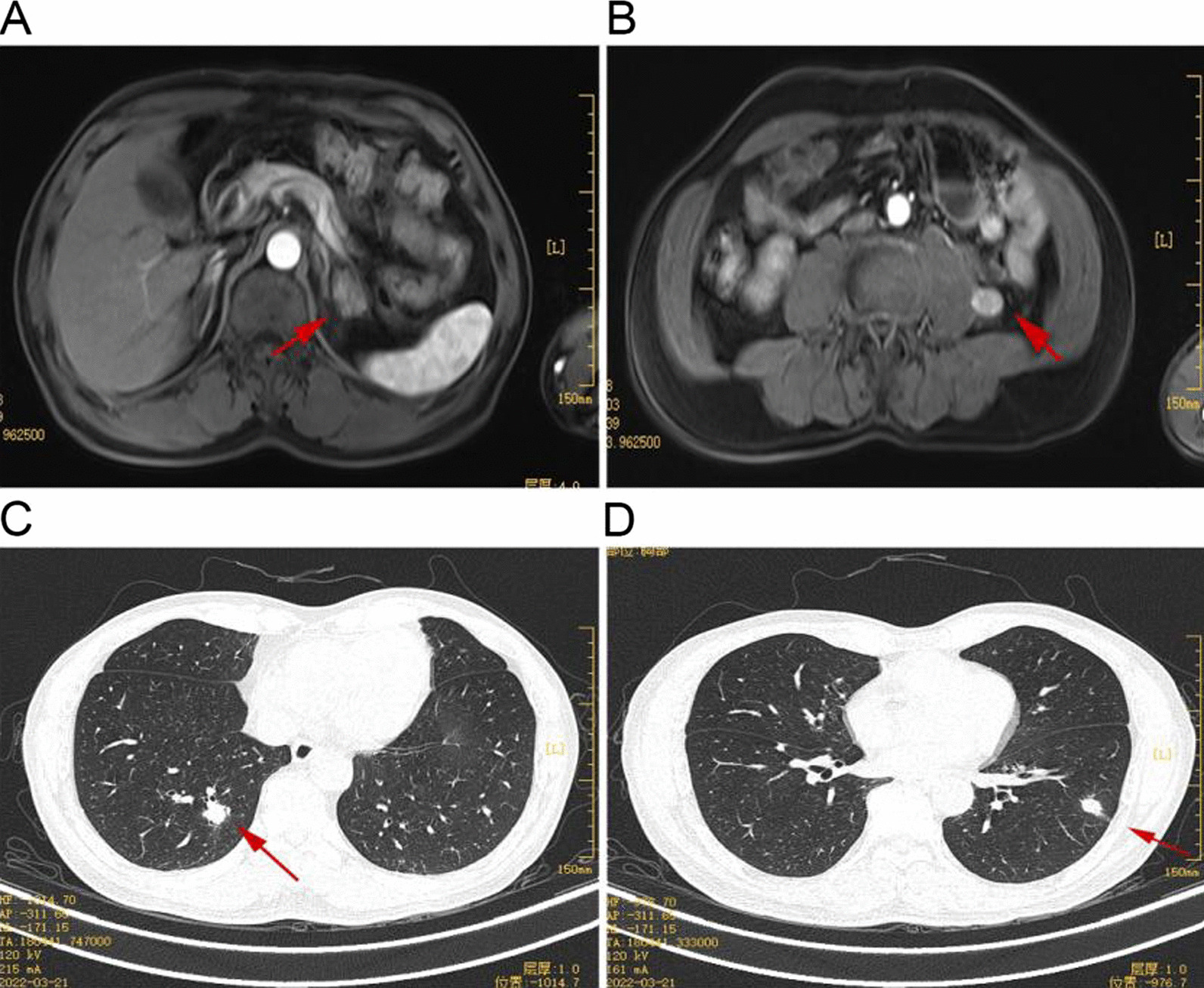
**A**, **B** Abdominal MRI images show a mass in the left adrenal region and a mass beside the left psoas muscle, considered a local tumor recurrence. **C**, **D** The chest CT investigations show multiple nodule shadows in both lungs, considering distant metastasis of the tumor

After the initial three months of surgery during follow-up, no tumor and metastasis recurrence in other parts of the patient were observed in the surgical area. Subsequently, the patient had no significant complaints during the 6-month postoperative follow-up. Nevertheless, the abdominal MRI showed nodular shadows on the left side of the adrenal gland and the left side of the lumbar muscle, with sizes of 2.5 * 2.1 cm^2^ and 1.5 * 1.3 cm^2^, respectively. In addition, the chest CT revealed multiple lung nodules, which were confirmed to be tumor metastasis (Fig. 4).

**Fig. 5:**
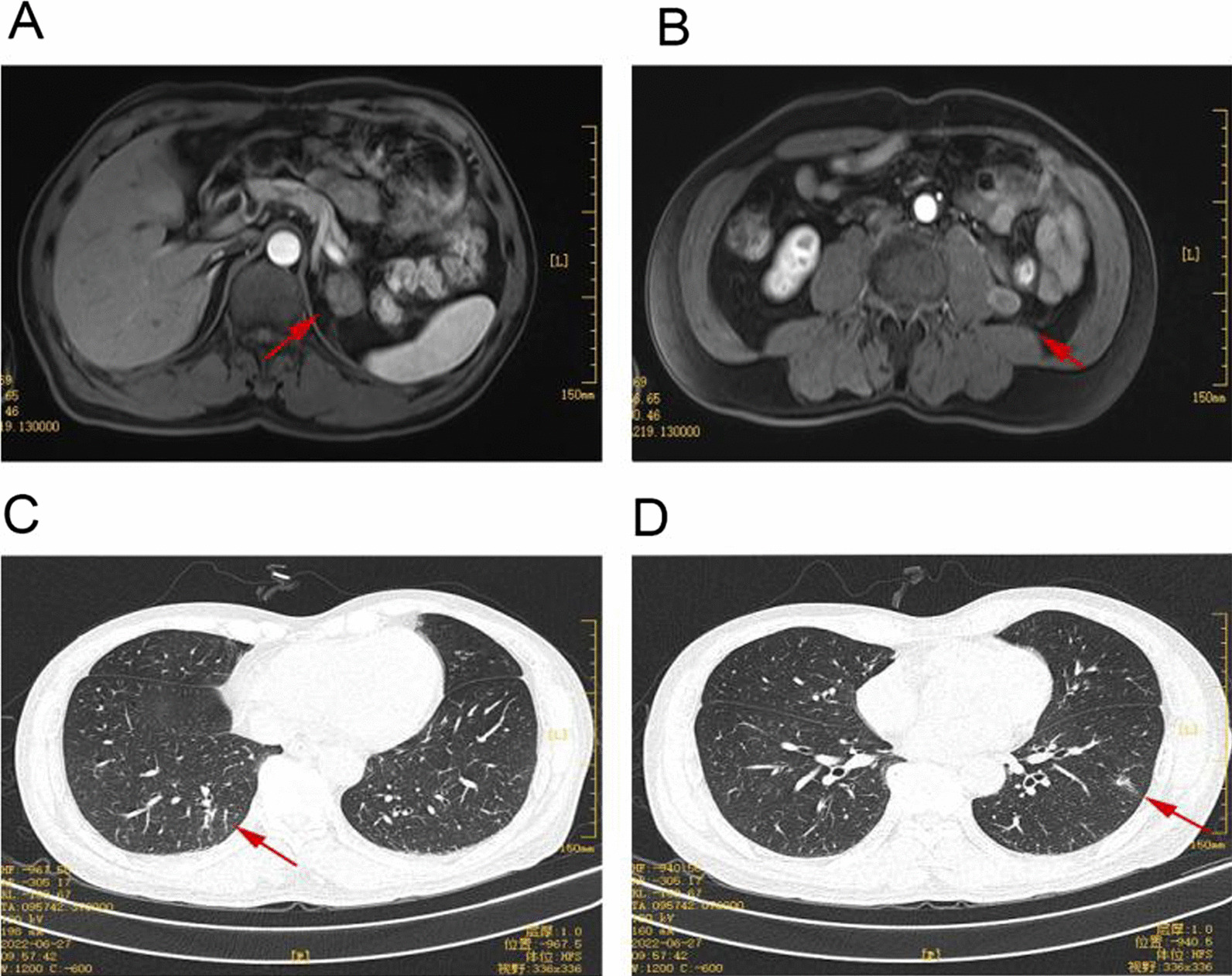
**A**, **B** Abdominal MRI images show the tumor in the left adrenal region and near the left psoas muscle with the same sizes. **C**, **D** The chest CT images show smaller shadows of multiple nodules in both lungs than before

While compiling this case report, the patient received the targeted therapy with anlotinib, administered for three months. Further, the chest CT results after treatment indicated that the metastatic tumor size was significantly reduced compared to before treatment. Nonetheless, the abdominal MRI indicated no significant change in the size of retroperitoneal tumors (Fig. 5).

## Discussion and conclusion

LPS is the most common histological type of retroperitoneal sarcoma (RPS), accounting for approximately 20% of all sarcomas in adults [4]. LPS subtypes include well-differentiated LPS (WDLPS), dedifferentiated LPS (DDLPS), myxoid LPS (MLPS), pleomorphic LPS (PLPS), and the newly described, myxoid pleomorphic LPS (MPLPS) [5]. In this context, several factors affect the prognosis of patients with RPLPS, such as tumor size, margin involvement after resection, and histological grade. Indeed, distant metastasis is often related to tumor size. A previous study indicated that a larger tumor size was associated with a higher five-year distant metastasis rate, in which a tumor size of over 20 cm would result in a five-year distant metastasis rate of 55–60% [6]. In addition, the margin involvement after resection affects the prognosis of the LPS disease. To demonstrate this aspect, a tendency of local recurrence was reported in cases with resection margin involvement [7]. A meta-analysis report in 2018 indicated that the histological subtype of LPS was the main prognostic factor for local recurrence and distant metastasis. Among various sarcomas, the local recurrence-free survival rate (RFS) of DDLPS and PLPS was the worst, while distant metastasis was the most common in PLPS and MLPS. Typically, the reports suggested that the lung was the leading site of distant metastasis [8]. Indeed, the surgical resection of a malignant tumor is the first choice for treating primary RPLPS. The ideal surgical treatment for primary RPLPS intends to completely remove the tumor and obtain a negative microscopic margin (R0). To date, no official consensus on the scope of surgical resection of RPLPS has been reported. However, a healthy 1-cm margin around the primary tumor can be considered a sufficient R0 resection, which is challenging. Due to the local aggressiveness of LPS in terms of metastasis, RPLPS usually grows very large, often involving many adjacent organs and structures. It should be noted that the most common difficulty during the sectioning is infiltrating through the tumor area to determine the retroperitoneal structure for conveniently controlling the major vessels of the resected organs.

In addition, it is highly challenging to distinguish the highly differentiated adipocytes in the tumor from retroperitoneal fat. The invasion of RPLPS into other tissues and organs is much more severe than the anticipation, including the highly differentiated LPS [9]. Therefore, it is complicated to determine the safe boundary of resection. In a case, the median survival of patients with complete resection of the margin was reported as 103 months, higher in patients with incomplete resection (18 months) [10]. It should be noted that incomplete resection seriously affects the prognosis of the disease. Despite the controversies, the significant conceptual progress in the surgical scope in recent years has gradually advocated achieving the goal of radical resection. The new concept of this surgical method, i.e., “extended resection or septal resection,“ relies on removing adjacent organs and structures (even if they are not infiltrated or affected by the tumors). In addition, this approach maximizes the possibility of negative microdissection margins and jointly removes organs or structures, including the kidney, colon, pancreas, spleen, psoas major, diaphragm, and all ipsilateral retroperitoneal adipose tissues from the diaphragm to one side of the tumor’s iliac blood vessel. Previous studies indicated that the local recurrence rate of patients receiving extended resection was reported to be lower than that of patients receiving standard resection [11, 12]. Nevertheless, extensive resection often could increase the risk of postoperative complications. For instance, simultaneous resecting of three or more organs might increase the chances of mortality [13]. However, in some instances, it has been reported that complications in patients caused by the combined resection of multiple organs showed no substantial effect on the overall survival (OS) rate [13]. Therefore, the current surgical strategy of our research team for RPLPS involved the joint removal of some adjacent organs according to the concept of " extended resection or septal resection”. Notably, we have achieved en-bloc resection as far as possible to preserve the patient’s core organs and strive for negative microsurgical margins to prevent local recurrence and improve the survival rate.

Indeed, surgical resection is still the first choice for locally recurrent RPLPS. A retrospective study of recurrent RPLPS showed that the average recurrence time of DDLPS was the earliest of all subtypes, i.e., 10.8 months from the first diagnosis [14]. Reportedly, local recurrence occurs would influence the growth rate of the tumor, affecting the survival and prognosis. Although proper surgical treatment has been carried out, the growth rate exceeding 0.9 cm per month is still an ominous sign [15]. In the case of a high risk of metastasis and difficulty to remove, surgical removal of tumors as soon as possible is inevitable for tumors growing too fast or for a high grade at specific locations. In addition, it is required and feasible to closely observe low-grade locally recurrent tumors that grow slowly or continue to grow.

Although their undeniable beneficial effects in some patients, some traditional therapeutic modalities, such as radiotherapy and chemotherapy, are assumed to have controversial effectiveness in treating RPLPS. A systematic review and meta-analysis showed that surgery combined with radiotherapy significantly improved the OS of patients compared with surgery alone. The median RFS was significantly prolonged in both preoperative and postoperative radiotherapy groups [16]. Since 1974, doxorubicin has been applied for treating soft tissue sarcoma. Its single-drug chemotherapy and combined chemotherapy with ifosfamide have still been prescribed as the first-line standard chemotherapy scheme for advanced or metastatic soft tissue sarcoma. However, the substantial therapeutic effects are far from satisfactory [17, 18]. Previous studies demonstrated that WDLPS and DDLPS responded poorly to chemotherapy or radiotherapy, while MLPS and PLPS subtypes showed excellent responses [8, 19, 20]. Accordingly, patients with recurrent RPLPS suitable for surgical resection should first receive induction radiotherapy, while patients insensitive to radiation should receive neoadjuvant chemotherapy [10, 21, 22]. Notably, chemotherapy was not recommended for patients who recovered from surgery for recurrent intraperitoneal LPS in the case of a confirmed surgical margin of R0 [21, 23]. In the R1 or R2 margin case, chemotherapy was superior to regional radiotherapy or brachytherapy [21]. Carboni et al. further confirmed that chemotherapy was a palliative treatment for advanced or metastatic diseases [24]. For unresectable or metastatic RPLPS, partial resection showed slight clinical benefits, which could attenuate some symptoms and partially improve the quality of life. Therefore, it should be noted that this combinatorial treatment modality should be carefully selected.

Targeted therapy is another option for treating recurrent LPS. Anlotinib, a second-line treatment strategy for unresectable or advanced LPS, improves progression-free survival (PFS) and OS in patients with advanced soft tissue sarcoma [25, 26]. In a case, it was demonstrated that the selective cyclin-dependent kinase 4 (CDK4) gene was amplified in 90% of WDLPS and DDLPS [27]. In this context, the latest recommended targeted drug, the CDK4 inhibitor named palbociclib, can be considered the first-line treatment of specific types of unresectable late soft-tissue sarcomas. Previous studies showed that palbociclib could improve PFS in patients with RPLPS, achieving long-term stability in some patients with WDLPS or DDLPS [28, 29].

In the present case, the patient showed signs of tumor recurrence after radical surgery, considering metastatic and advanced RPLPS and indicating no requirement for secondary surgery. Due to the poor response of DDLPS to radiotherapy and chemotherapy, and the high toxicity of radiotherapy and chemotherapy, we decided to administer targeted therapy with anlotinib. The reexamination results after three-month targeted treatment with anlotinib showed that the size of multiple lung metastases was significantly decreased. In contrast, the size of the recurrent retroperitoneal tumors was not significantly changed. In conclusion, no evidence of tumor progression was observed, and the patient’s condition was under control.

RPLPS is a rare malignant tumor, considering surgery as its first-line treatment. Due to the common postoperative recurrence, close imaging follow-up examinations are necessary at regular intervals. In addition, it is also necessary to reasonably select different treatment methods, such as surgery, radiotherapy, chemotherapy, targeted therapy, and immunotherapy. Therefore, multidisciplinary team cooperation involving surgery, oncology, radiation oncology, and other medical specialties is essential for effectively treating such case.

## Data Availability

All data generated or analyzed during this study are included in this published article.

## Abbreviations

CDK4: Cyclin-dependent kinase 4
CT: Computed tomography
DDLPS: Dedifferentiated liposarcoma
LPS: Liposarcoma
MLPS: Myxoid liposarcoma
MPLPS: Myxoid pleomorphic liposarcoma
MRI: Magnetic resonance imaging
OS: Overall survival
PFS: Progression-free survival
PLPS: Pleomorphic liposarcoma
RFS: Recurrence-free survival rate
RPLPS: Retroperitoneal liposarcoma
WDLPS: Well-differentiated liposarcoma
